# Protocol for functional profiling of patient-derived organoids for precision oncology

**DOI:** 10.1016/j.xpro.2024.102887

**Published:** 2024-02-16

**Authors:** Niloofar Nemati, Nina Boeck, Giorgia Lamberti, Rebecca Lisandrelli, Zlatko Trajanoski

**Affiliations:** 1 Biocenter, Institute of Bioinformatics, Medical University of Innsbruck, 6020 Innsbruck, Austria

**Keywords:** Cancer, Sequencing, RNAseq, Organoids, Systems biology

## Abstract

Functional precision oncology—a strategy based on perturbing primary tumor cells from cancer patients—could provide a road forward for personalized treatment. Here, we present a comprehensive protocol covering generation and culture of patient-derived colorectal organoids, isolation and expansion of tumor-infiltrating lymphocytes (TILs), and isolation and culture of peripheral blood mononuclear cells (PBMCs). With this protocol, samples fulfilling the demands for performing multi-omics analysis, e.g., RNA sequencing (RNA-seq), whole-exome sequencing (WES), single-cell RNA sequencing (scRNA-seq), and (phospho-)proteomics, can be generated.

For complete details on the use and execution of this protocol, please refer to Plattner et al. (2023).^1^

## Highlights

• Specimen preparation for molecular and genomic characterization of primary tumor
• Biobank generation of patient-derived CRC organoids for potential drug screenings
• Preservation of cancer and immune cells for advanced *ex vivo* culture techniques
• Perturbation experiments for reconstruction of kinase networks for precision oncology

## Abstract (editor highlights)

Publisher’s note: Undertaking any experimental protocol requires adherence to local institutional guidelines for laboratory safety and ethics.

## Graphical abstract

**Figure:**
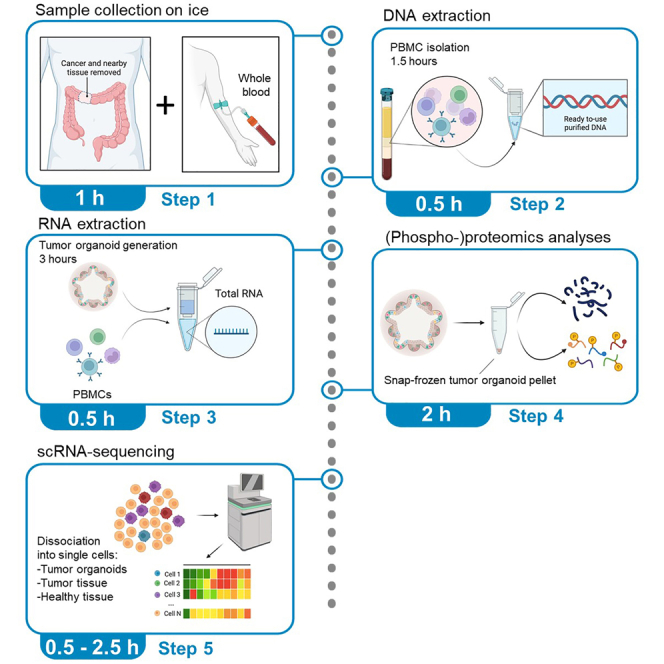

## Before you begin

This protocol describes in detail the preparation of different specimens derived from resected colorectal cancer tissue. By using healthy and tumor tissue as well as autologous blood samples for RNA-seq and WES analysis, the primary tumor is characterized. Proteome activity and phosphoproteomic data can be generated by using patient-derived organoids for perturbation experiments with kinase inhibitors. Furthermore, this protocol includes the isolation of TILs, which are important components of the tumor microenvironment (TME).

Before sample collection, prepare the necessary media, buffers and digestion mixes, in order to process the specimens as fast as possible. Some reagents and media can be prepared in advance and stored, some need to be prepared fresh and need to be used up immediately. Perform the whole protocol under sterile conditions and use a cell-culture hood. RNA and DNA can be extracted under a fume hood.

### Institutional permissions

Colorectal cancer tissue samples were procured from individuals who underwent surgery at the Tirol Kliniken in Innsbruck, Austria. The Ethical Committee at the Medical University of Innsbruck granted approval for the study, and written informed consent was obtained from the patients prior to surgical sampling. Any leftover tissue samples not designated for routine pathological examination were promptly transported to the laboratory for cell isolation within a maximum time frame of 6 h post-surgery.

Prior to initiating the steps described in this protocol, assure you have obtained the required institutional approval to work with patient-derived samples.

***Note:*** The laboratory carrying out tissue processing and cell isolation must be authorized with Biosafety Level 2 clearance and equipped with Laminar Flow cabinets. Given that primary human surgical materials may carry infectious risks in the absence of testing for all relevant pathogens (HCV, HBV, HIV, etc.), strict adherence to institutional biosafety regulations is mandatory. This includes the proper usage of personal protective equipment (e.g., lab coats, gloves), thorough decontamination of surfaces and appropriate waste disposal procedures.

### Preparation for sample collection

**Timing: 45 min to 1 h**

**Figure 1:**
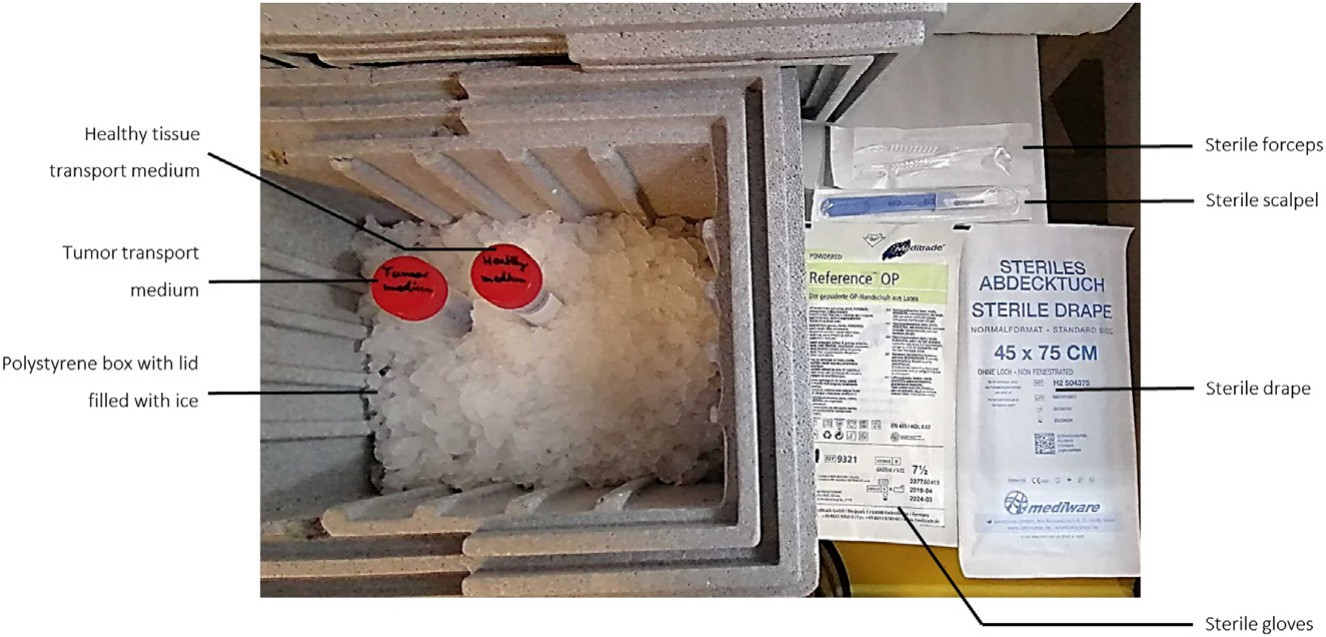
Transport box for tissue collection from the pathologist Items needed for pickup of the resected specimen from the pathologist. Keep transportation media, patient tissues and blood samples on ice and process immediately.

**CRITICAL:** Prepare transport box for tissue collection in the morning of the day of surgery in order to pick up the specimen as fast as possible (Figure 1).

1. Prepare all media (collection and culture media, wash media and digestion mixes) according to the tables in the ‘materials and equipment’ section in a sterile environment and store properly.

***Note:*** Prepare aliquots of media, small molecules and enzymes as indicated in the ‘material and equipment’ section, e.g., to avoid repeated freeze-thaw cycles.

2. Prepare the following reagents and keep or thaw on ice until use:
  a. Tumor tissue collection medium.
  b. Healthy tissue collection medium.
  c. Wash buffer.
  d. Crypts isolation medium.
  e. Fetal Bovine Serum (FBS) (thaw).
  f. Red Blood Cell (RBC) Lysis buffer (1×).
  g. DPBS.
3. If needed, reconstitute the following reagents according to manufacturer’s recommendations and thaw on ice:
  a. Liberase DH (reconstitute).
  b. DNase I (reconstitute).
  c. Geltrex.
4. Warm up the following media and reagents to 37°C:
  a. Advanced DMEM/F-12.
  b. Tumor organoid medium.
  c. Healthy organoid medium.
  d. PBMC culture medium.
  e. TILs isolation medium.
  f. DPBS.
5. Switch on the following instruments.
  a. Centrifuges (at 20°C and at 4°C).
  b. Thermostable shaker, set temperature to 37°C.
  c. Water bath, set temperature to 37°C.
  d. GentleMACS Octo Dissociator with heaters.
6. Assure the following media are adjusted to 20°C ± 2°C before usage.
  a. Lymphocyte separation medium.
  b. DPBS.
  c. PBMCs cryopreservation medium.
  d. TILs cryopreservation medium.

## Key resources table

**Table:** 

REAGENT or RESOURCE	SOURCE	IDENTIFIER
**Antibodies**		

Anti-human CD45-PE (HI30), used as 1:16 dilution	BioLegend	Cat# 304008; RRID: AB_314396
Anti-human CD4-BV421 (RPA-T4), used as 1:20 dilution	BD Biosciences	Cat# 562424; RRID: AB_11154417
Anti-human CD8-FITC (G42-8), used as 1:40 dilution	BD Biosciences	Cat# 551347; RRID: AB_394159
Anti-human EpCAM-APC (9C4), used as 1:16 dilution	BioLegend	Cat# 324207; RRID: AB_756081
7-Aminoactinomycin D, used as 1:20 dilution	BD Biosciences	Cat# 559925; RRID: AB_2869266

**Biological samples**		

Resected colorectal tumors and adjacent healthy colorectal tissue samples, untreated	Tirol Kliniken Innsbruck	N/A
Tumor-infiltrated lymphocytes from resected colorectal tumors	Tirol Kliniken Innsbruck	N/A
Peripheral blood mononuclear cells from patients undergoing colorectal tumor resections	Tirol Kliniken Innsbruck	N/A
Peripheral blood mononuclear cells from healthy donors, bloodbank	Tirol Kliniken Innsbruck, 1× LRSC per request	N/A
HA-R-Spondin 1-Fc 293T cells	AMSBIO/293T-HA-Rspo-Fc cells	Cat# 3710-001-01; Home made
Cell-line for production of Noggin	Hubrecht Institute/HEK293-mNoggin-Fc cells	Home made

**Chemicals, peptides, and recombinant proteins**		

Advanced DMEM/F-12	Life Technologies	Cat# 12634-010
StemPro hESC SFM	Gibco	Cat# A1000701
GlutaMAX supplement	Gibco	Cat# 35050061
Penicillin-Streptomycin	Sigma-Aldrich	Cat# P4333
HEPES buffer	Sigma-Aldrich	Cat# H0887
B27 supplement	Life Technologies	Cat# 17504044
N-acetyl-L-cysteine BioReagent	Sigma-Aldrich	Cat# A9165-5G
A83_01	Tocris	Cat# 2939
SB202190	Sigma-Aldrich	Cat# S7067-5MG
Recombinant human EGF	PeproTech	Cat# AF-100-15
Primocin	InvivoGen	Cat# ant-pm-2
Y-27632 dihydrochloride	TargetMol/Hölzel Diagnostika Handels GmbH	Cat# TMO-T1725
Wnt surrogate Fc fusion protein	U-Protein Express BV	Cat# N001
Nicotinamide	Sigma-Aldrich	Cat# N0636
Gastrin I, human	Sigma-Aldrich	Cat# G9020-250UG
Prostaglandin E2	Cayman/Biomol	Cat# Cay-14010
Geltrex LDEV-free reduced growth factor BMM	Fisher Scientific	Cat# A1413202
L-glutamine, 200 mM	Sigma-Aldrich	Cat# G7513-100mL
Red blood cell lysis buffer, RBC lysis buffer (10×)	BioLegend/Biozym	Cat# B420301
Recovery cell culture freezing medium	Invitrogen	Cat# 12648-010
Trypsin-EDTA	Sigma-Aldrich	Cat# T4174
RPMI 1640 medium (ATCC modification)	Sigma-Aldrich	Cat# R0883
Heat-inactivated human AB serum	Sigma-Aldrich	Cat# H3667
Fetal bovine serum (FBS), sterile-filtered	Sigma-Aldrich	Cat# F7524
Trypan blue solution	Sigma-Aldrich	Cat# T8154
Proleukin, Aldesleukin (18 × 10^6^ IU)	Novartis	N/A
Lymphocyte separation medium	Capricorn Scientific	Cat# LSM-A
TrypLE Express enzyme (1×)	Invitrogen	Cat# 12604013
Dulbecco’s phosphate-buffered saline, without calcium and magnesium chloride, sterile-filtered, DPBS	Sigma-Aldrich	Cat# D8537
Ultrapure water (type 1), bottled	Sartorius	N/A
Ethanol for molecular biology, ≥99.8% purity	VWR International	Cat# 1.08543.0250
Bovine serum albumin (BSA)	Sigma-Aldrich	Cat# A6003
Liberase DH, research grade	Roche	Cat# 5401054001
DNAse I, grade II, from bovine pancreas	Roche/Sigma-Aldrich	Cat# 10104159001
DMEM, high glucose, pyruvate, no glutamine	Gibco	Cat# 21969-035
Cell recovery solution	Corning	Cat# 7340107
β-Mercaptoethanol	Sigma-Aldrich	Cat# 63689
Dimethyl sulfoxide for molecular biology (DMSO)	Sigma-Aldrich	Cat# D8418
UltraPure 0.5 M EDTA, pH 8.0	Invitrogen	Cat# 15575-020
Zeocin	Life Technologies	Cat# R25005
Geneticin G418	Sigma-Aldrich	Cat# A1720
Accutase cell detachment solution	BioLegend	Cat# B423201

**Critical commercial assays**		

PureLink Genomic DNA Mini Kit	Thermo Fisher Scientific	Cat# K182001
RNeasy Plus Mini Kit	QIAGEN	Cat# 74134
QIAshredder	QIAGEN	Cat# 79654
RNase-free DNase set	QIAGEN	Cat# 79254
Genomic DNA Clean & Concentrator	Zymo Research	Cat# D4010

**Software and algorithms**		

FlowJo v10.7.2	FlowJo	www.flowjo.com
GraphPad Prism 9	GraphPad	www.graphpad.com

**Other**		

15 mL centrifuge tube	Starlab	Cat# E1415-0100
50 mL centrifuge tube	Sarstedt	Cat# 62.547.254
CytoOne 6 cm Petri dish, uncoated	Starlab	Cat# CC7672-3359
Stainless steel surgical blades, sterile, Nr. 22	VWR International GmbH	Cat# 233-5484
Cell culture plates, 6-well plate, flat bottom	Sarstedt	Cat# 83.3920
Cell scraper, 24 cm, size: S	Sarstedt	Cat# 83.3950
Cell scraper, 25 cm, size: M	Sarstedt	Cat# 83.1830
Corning cell lifter	Merck	Cat# CLS3008-100EA
pluriStrainer, 100 μm strainer, sterile	pluriSelect	Cat# 43-50100-51
pluriStrainer, 400 μm strainer, sterile	pluriSelect	Cat# 43-50400-03
Connector ring	PluriSelect	Cat# 41-50000-03
Cryogenic vials	Starlab	Cat# E3090-6222
Cell culture plates, 24-well, flat bottom	Sarstedt	Cat# 83.3922.005
Cell culture plates, 6-well	Sarstedt	Cat# 83.3920
Cell culture flasks, T-25	Sarstedt	Cat# 83.1810.002
Cell culture flasks, T-75	Sarstedt	Cat# 83.3911.002
Cell culture flasks, T-175	Sarstedt	Cat# 83.3912.002
Tissue culture dish, standard 100∗200 mm	Sarstedt	Cat# 83.3902
BD Vacutainer 10 mL plastic blood collection tubes with sodium heparin	Fisher Scientific	Cat# 02-689-6
Mediware, disposable scalpels, stainless steel, sterile (for tissue dissection)	medizinbedarf.at	Cat# I1 09
Mediware, disposable forceps, sterile (for tissue dissection)	medizinbedarf.at	Cat# H7 301
Mediware pre-powdered gloves	medizinbedarf.at	Cat#9321
Mediware sterile drapes	medizinbedarf.at	Cat#H2 50
Hemocytometer	N/A	N/A
Liquid nitrogen freezer	N/A	N/A
Freezer, −80°C	N/A	N/A
Fridge, 4°C	N/A	N/A
Axio ZEISS ZEN microscope	ZEISS	N/A
FACS Fortessa, Flow Cytometer	BD	N/A
gentleMACS Octo Dissociator with heaters	Miltenyi Biotec	Cat# 130-096-427
Freezing container, Mr. Frosty, Nalgene	VWR International GmbH	Cat# 479-3200
Integra Pipetboy acu 2	VWR	Cat# 612-0926
Cell counting slides for TC10, dual chamber	Bio-Rad	Cat# 145-0016
TC-10 automated cell counter	Bio-Rad	Cat# 145-0001
Safe seal reaction tube, 1.5 mL, polypropylene	Sarstedt	Cat# 72.706
Safe seal reaction tube, 2 mL, polypropylene	Sarstedt	Cat# 72.695.500
TubeOne 1.5 mL natural flat microcentrifuge tubes, free of detectable RNase, DNase	Starlab	Cat# S1615-5500
Safe seal reaction tube, 2 mL, polypropylene PCR performance tested, free of detectable RNase, DNase	Sarstedt	Cat# 72.695.400
Filter tips, 10 μL, sterile	Starlab	Cat# S1121-3810
Filter tips, 20 μL, sterile	Starlab	Cat# S1120-1810
Filter tips, 100 μL, sterile	Starlab	Cat# S1123-1840
Filter tips, 200 μL, sterile	Starlab	Cat# S1120-8810
Filter tips, 1000 μL, sterile	Starlab	Cat# S1126-7810
Water bath	N/A	N/A
Heat block	N/A	N/A
Microcentrifuge machine	N/A	N/A
Cell strainer, 40 μm, Corning	VWR	Cat# 734-0002
Serological pipette, 5 mL	Sarstedt	Cat# 86.1253.001
Serological pipette, 10 mL	Sarstedt	Cat# 86.1254.001
Serological pipette, 25 mL	Sarstedt	Cat# 86.1685.001
Serological pipette, 50 mL	Sarstedt	Cat# 86.1256.001
gentleMACS Octo Dissociator with heaters, C tubes	Miltenyi Biotec	Cat# 130-093-237
Stericup	Millipore	Cat# SCGVU05RE
IKA KS 4000 i Thermostable shaker	Fisher Scientific	Cat# 10408643
Stuart rotator, SB3	VWR	Cat# 445-2101
KERN analytical balance	Merck	Cat# Z741091
Vacuum pump, Vacusafe aspiration system with integrated pump	INTEGRA Biosciences	Cat# 158320

## Materials and equipment

### Culture media recipes and digestion mixes

**Table:** StemPro hESC SFM complete medium

Reagent	Final concentration	Amount
DMEM/F-12 with GlutaMAX medium	N/A	500 mL
Bovine serum albumin (BSA)	25%	40 mL
StemPro hESC Supplement (50×)	1×	10 mL
Penicillin-Streptomycin (100×)	1×	5 mL
Primocin (500×)	1×	1 mL
Total	N/A	556 mL

Prepare in a sterile environment, store at 4°C for up to 1 month. Prepare aliquots of 18 mL and keep on ice until further use.

**Table:** Tumor tissue collection medium

Reagent	Final concentration	Amount
StemPro hESC SFM complete medium	N/A	18 mL
Fetal Bovine Serum (FBS) (10×)	1×	2 mL
Total	N/A	20 mL

Prepare in a sterile environment, store at 4°C for up to 1 month. Keep aliquots of 20 mL on ice until further usage.

**Table:** Healthy tissue collection medium

Reagent	Final concentration	Amount
DPBS	1×	500 mL
Penicillin-Streptomycin (100×)	1×	5 mL
Primocin (500×)	1×	1 mL
Total	N/A	506 mL

Prepare in a sterile environment, store at 4°C for up to 1 month. Prepare aliquots of 20 mL and keep on ice until further usage.

**Table:** Wash buffer

Reagent	Final concentration	Amount
DPBS	1×	500 mL
Penicillin-Streptomycin (100×)	1×	5 mL
Primocin (500×)	1×	1 mL
Total	N/A	506 mL

Prepare in a sterile environment, store at 4°C for up to 1 month. Prepare aliquots of 20 mL and keep on ice until further usage.

**Table:** Crypts isolation medium

Reagent	Final concentration	Amount
EDTA (0.5 M)	10 mM	200 μL
DPBS	1×	9.8 mL
Total	N/A	10 mL

Prepare in a sterile environment, store at 4°C and use immediately on the day of tissue processing.

**Table:** Advanced DMEM/F-12

Reagent	Final concentration	Amount
Advanced DMEM/F-12	N/A	500 mL
GlutaMAX (100×)	1×	5 mL
Penicillin-Streptomycin (100×)	1×	5 mL
HEPES (1 M)	10 mM	5 mL
Total	N/A	515 mL

Prepare in a sterile environment, store at 4°C for up to 4 weeks. Prepare aliquots of 50 mL for warming up to 37°C.

**Table:** PBMC culture medium

Reagent	Final concentration	Amount
RPMI 1640 (ATCC modification)	1×	500 mL
Fetal Bovine Serum (FBS)	10%	50 mL
Penicillin-Streptomycin	1%	5 mL
L-Glutamine (200 mM)	2 mM	5 mL
Total	N/A	560 mL

Prepare in a sterile environment, store at 4°C for up to 4 weeks. Prepare aliquots of 50 mL for warming up to 37°C.

**Table:** PBMC freezing medium

Reagent	Final concentration	Amount
PBMC culture medium	45%	4.5 mL
Fetal Bovine Serum (FBS)	45%	4.5 mL
DMSO	10%	1 mL
Total	N/A	10 mL

Prepare in a sterile environment, store at −20°C for up to 3 months.

**Table:** Tumor organoid culture medium

Reagent	Final concentration	Amount
Advanced DMEM/F-12	N/A	33 mL 700 μL
R-spondin1 conditioned medium (see notes)	20%	10 mL
Noggin conditioned medium (see notes)	10%	5 mL
B27 (50×)	1×	1 mL
N-Acetylcysteine (500 mM)	1.25 mM	125 μL
A83-01 (500 μM)	500 nM	50 μL
SB202190 (30 mM)	10 μM	16.6 μL
hEGF (0.5 mg/mL	50 ng/mL	5 μL
Primocin (50 mg/mL)	100 μg/mL	100 μL
Y-27632 (100 mM)	10 μM	5 μL
Total	N/A	50 mL

Prepare fresh in a sterile environment, store at 4°C and use up within 10 days.

**Table:** Healthy organoid culture medium

Reagent	Final concentration	Amount
Advanced DMEM/F-12	N/A	33 mL 200 μL
Wnt Fc Fusion Protein (5 μM)	0.5 nM	5 μL
R-spondin 1 conditioned medium (see notes)	20%	10 mL
Noggin conditioned medium (see notes)	10%	5 mL
B27 (50×)	1×	1 mL
N-Acetylcysteine (500 mM)	1.25 mM	125 μL
A83-01 (500 μM)	500 nM	50 μL
SB202190 (30 mM)	10 μM	16.6 μL
Gastrin (100 μM)	10 nM	5 μL
hEGF (0.5 mg/mL	50 ng/mL	5 μL
Primocin (50 mg/mL)	100 μg/mL	100 μL
Y-27632 (100 mM)	10 μM	5 μL
Prostaglandin PGE2 (100 μM)	10 nM	5 μL
Total	N/A	50 mL

Prepare fresh in a sterile environment, store at 4°C and use up within 10 days.

**Table:** Small molecule preparation for tumor and healthy organoid culture medium

Reagent	Preparation	Storage / aliquots
N-Acetylcysteine	500 mM stock in H2O	−20°C for up to one year / 500 μL aliquots
Nicotinamide	1 M stock in DPBS	−20°C for up to one year / 2 mL aliquots
Gastrin	100 μM stock in DPBS	−20°C for up to one year / 20 μL aliquots
Y-27632	100 mM stock in DMSO	−20°C for up to one year / 20 μL aliquots
A83-01	500 μM stock in DMSO	−20°C for up to one year / 200 μL aliquots
SB202190	30 mM stock in DMSO	−20°C for up to one year / 75 μL aliquots
hEGF	0.5 mg/mL in DPBS with 0.1% BSA	−20°C for up to one year / 20 μL aliquots
B27	stock solution is provided at 50× concentration	−20°C for up to one year / 2 mL aliquots
Primocin	solution is provided at a stock concentration of 50 mg/mL	4°C for 6 months or at −20°C for long-term storage / 400 μL aliquots
Prostaglandin PGE2	100 μM stock in DMSO	−20°C ≥ 2 years / 20 μL aliquots

**Table:** TILs freezing medium

Reagent	Final concentration	Amount
Fetal Bovine Serum (FBS)	90%	9 mL
DMSO	10%	1 mL
Total	N/A	10 mL

Prepare in a sterile environment, store at −20°C for up to 3 months.

Alternative for PBMC cryopreservation medium: this freezing medium can be used for cryopreservation of PBMCs as well.

**Table:** TILs isolation medium

Reagent	Final concentration	Amount
RPMI 1640 (ATCC modification)	1×	500 mL
Human Serum	7.5%	37.5 mL
Penicillin-Streptomycin	1%	5 mL
L-Glutamine (200 mM)	2 mM	5 mL
Interleukin-2 (IL-2)	1000 U/mL	N/A
Total	N/A	547.5 mL

Prepare in a sterile environment, store at 4°C for up to 4 weeks, without addition of IL-2. Prepare aliquots of 50 mL for warming up to 37°C.

**Table:** TILs expansion medium

Reagent	Final concentration	Amount
RPMI 1640 (ATCC modification)	1×	500 mL
Human Serum	10%	50 mL
Penicillin-Streptomycin	1%	5 mL
L-Glutamine (200 mM)	2 mM	5 mL
Interleukin-2 (IL-2)	3000 U/mL	N/A
Anti-CD3 (OKT-3)	30 ng/mL	N/A
Feeder cells (irradiated PBMCs)	100×	N/A
Total	N/A	560 mL

Prepare in a sterile environment, store at 4°C for up to 4 weeks, without addition of IL-2, anti-CD3 and feeder cells. Prepare aliquots of 50 mL for warming up to 37°C.

**Table:** Red Cell Blood Lysis Buffer Solution (RBC Lysis Buffer)

Reagent	Final concentration	Amount
RBC Lysis Buffer (10X)	1×	1 mL
Deionized water	N/A	9 mL
Total	N/A	10 mL

Prepare fresh and in a sterile environment, use immediately. Do not store 1× RBC Lysis Buffer.

**Table:** Tissue digestion medium

Reagent	Final concentration	Amount
DMEM	1×	500 mL
Penicillin-Streptomycin	1%	5 mL
L-Glutamine (200 mM)	2 mM	5 mL
Total	N/A	510 mL

Prepare in a sterile environment, store at 4°C for up to 4 weeks.

**Table:** Organoid digestion mix

Reagent	Final concentration	Amount
StemPro hESC SFM complete medium	1×	10 mL
Liberase DH	25 ng/mL	N/A
Total	N/A	10 mL

Prepare in a sterile environment and use up immediately. Do not store. Add enzyme to the StemPro hESC SFM complete medium before warming up to 37°C, because the optimal working temperature for most enzymes is 37°C.

**Table:** Tissue digestion mix

Reagent	Final concentration	Amount
Tissue digestion medium	1×	10 mL
Liberase DH	25 ng/mL	N/A
DNase I	10 mg/mL	N/A
Total	N/A	10 mL

Prepare in a sterile environment and use up immediately. Do not store. Add enzymes to the Tissue digestion medium before warming up to 37°C, because the optimal working temperature for most enzymes is 37°C.

## Step-by-step method details

### Isolation of peripheral blood mononuclear cells (PBMCs) from whole blood

**Timing: 1.5 h**

This section describes how to isolate mononuclear cells (PBMCs) from whole blood using density gradient centrifugation. The blood was drawn in 10 mL Sodium Heparin vacutainer anti-coagulation tubes. The blood sample should be processed as soon as possible after collection, ideally within 2–3 h (troubleshooting 1). Expect reduced cell recovery when sample processing is delayed and/or slow. The isolated PBMCs can be used directly or cryopreserved for future usage.

1. Prepare media and buffers specified in this segment before processing the blood:
  a. Ensure Lymphocyte separation media and DPBS are adjusted to 20°C ± 2°C.
  b. Thaw a 10 mL aliquot of PBMC cryopreservation medium.
  c. Cool down the centrifuge to 4°C.
  d. Label cryovials with intended cell number to be frozen, date and patient ID.
  e. Adjust a Mr. Frosty freezing container to 20°C ± 2°C and assure it is filled with the required amount of Isopropyl alcohol.

***Note:*** Keep the blood on ice from the time of blood draw and during transportation to the lab, as this drastically improves cell viability. Make sure not to exceed a maximum of one hour on ice to avoid blood coagulation.

***Note:*** Perform the entire protocol under aseptic conditions in a cell-culture hood and adhering to local regulations for biological hazards. When handling blood products, extensive precautions should be implemented to safeguard the health and safety of personnel.

**Figure 2:**
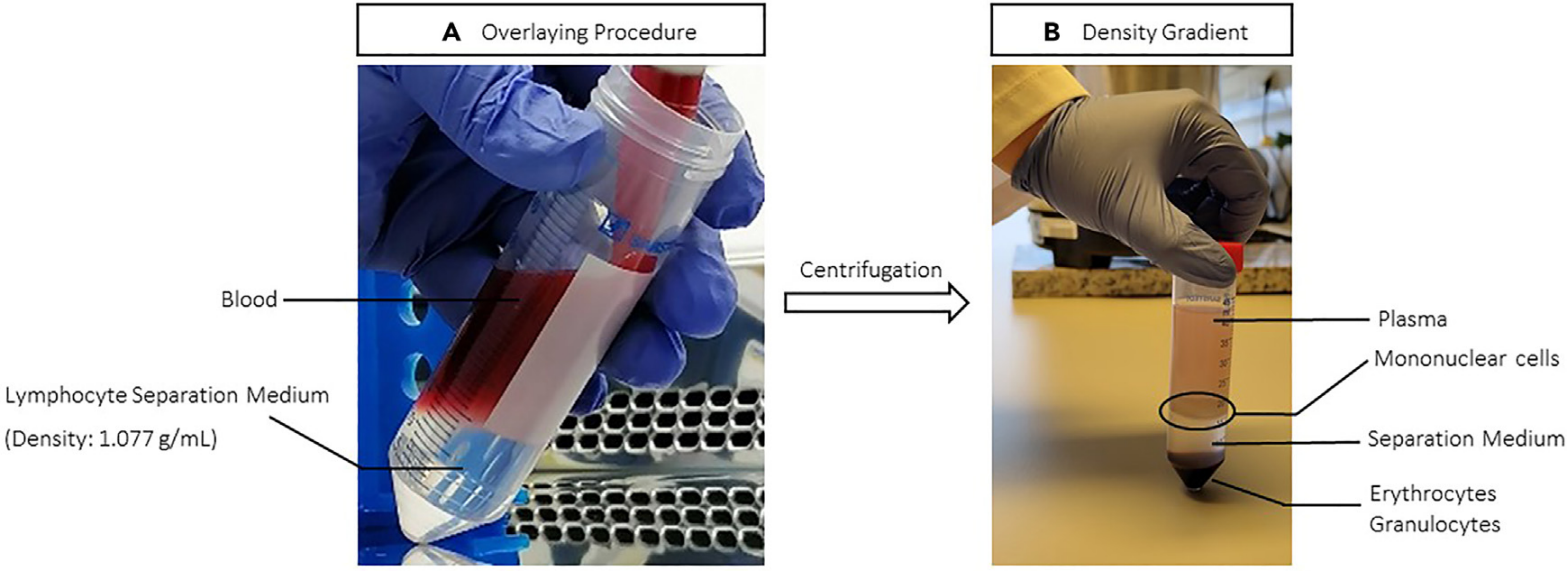
Workflow for isolation of PBMCs using density gradient centrifugation (A) Two distinct layers: blood diluted 1:2 with DPBS above Lymphocyte Separation medium before spin and density gradient after centrifugation (B). The marked circle indicates the layer of mononuclear cells.

2. Prepare two 50 mL centrifuge tubes with each 15 mL of Lymphocyte separation medium.
3. Transfer the blood from the anti-coagulation tubes into one 50 mL centrifuge tube.
4. Dilute the blood with DPBS by filling up the blood centrifuge tube to 50 mL and invert to mix thoroughly.
5. Gently layer 25 mL of the diluted blood on top of the separation medium of one of the two 50 mL centrifuge tubes (from Step 2) to form the density gradient. Repeat this procedure with the leftover 25 mL of the diluted blood and the second 50 mL centrifuge tube containing the separation medium (Figure 2A; troubleshooting 2).

**CRITICAL:** Hold the pipette against the tube wall, tilting the tube while filling it up and use the lowest speed setting on the pipette gun to not disturb the interface, ensuring a separate layer of medium and blood (Figure 2A). Immediately proceed with the centrifugation. The blood and separation medium will slowly blend if not subjected to centrifugation promptly.

6. Centrifuge at 500 × *g* for 30 min at 20°C using a swinging rotor with acceleration set at 3 and deceleration at 0 on the centrifuge.

**CRITICAL:** Keeping the break on, will interfere with the intactness of separate layers.

**CRITICAL:** Carefully move tubes following centrifugation not to disrupt the gradient.

***Note:*** After centrifugation, a whitish cloudy layer consisting of mononuclear cells will be found on top of the separation medium (Figure 2B).

7. Aspirate supernatant containing plasma and platelets up to 1 cm above PBMC layer.
8. Carefully collect PBMCs with a 10 mL serological pipet and place in a new 50 mL centrifuge tube.

***Note:*** Refrain from sucking up the separation medium and the layer containing erythrocytes.

9. Combine PBMCs from two 50 mL centrifuge tubes and fill up to 50 mL with DPBS.
10. Centrifuge at 450 × *g* for 10 min at 4°C.

**CRITICAL:** All following steps should be carried out with the cells placed on ice.

11. Aspirate supernatant and resuspend with 10 mL of cold DPBS.

***Note:*** Use a wide-bore pipette to not harm the cells.

12. Fill up the centrifuge tube to 50 mL with cold DPBS.
13. Centrifuge at 450 × *g* for 10 min at 4°C.
14. Resuspend in 1 mL DPBS for cell counting and viability determination.

***Note:*** Work fast and keep the cells on ice during the counting process.

***Note:*** Anticipate noticeable contamination from red blood cells, a concern mitigated following cryopreservation and subsequent assay procedures.

***Optional:*** Perform erythrolysis by incubating with 1× RBC lysis buffer for 10 min at 20°C ± 2°C and washing three times with DPBS to remove residual buffer.

15. Mix 10 μL of cell suspension with 90 μL Trypan blue (1:10 dilution) rapidly and inject 10 μL of the mix into a hemocytometer for cell counting using an inverted microscope. Trypan blue negative cells are considered viable cells.
16. After this step, continue by either using the cells directly for assays, for cryopreservation (see steps 17–23) or snap-freeze cell pellets for nucleic acid isolation (see steps 34–43).

### Cryopreservation and subsequent thawing of PBMCs

This section describes how to cryopreserve PBMCs for long-term storage and how to successfully thaw cells for culturing and performing downstream experiments.

#### Cryopreservation of PBMCs

**Timing: 30 min**

***Note:*** Pre-label cryovials and ensure availability of freezing containers.

**CRITICAL:** Prolonged exposure of cells to the freezing medium at 20°C ± 2°C may compromise cell viability and consequently, integrity upon thawing (troubleshooting 3).

***Note:*** Frozen PBMCs, intended for storage less than two weeks, can be maintained at −80°C. However, for extended storage beyond two weeks, it is advisable to store cryovials in the vapor phase of a liquid nitrogen tank.

**CRITICAL:** Work on ice.

17. Resuspend cells at 5–25 × 10^6^ viable cells/mL in ice-cold DPBS.
18. Transfer cells to a 15 mL tube with 10 mL of ice-cold DPBS.
19. Centrifuge at 405 × *g* for 5 min at 4°C.
20. Gently resuspend in 500 μL PBMC cryopreservation medium per aliquot.
21. Transfer 500 μL of the cell suspension to a cryovial.
22. Freeze at −80°C for 16–24 h in a Mr. Frosty freezing container.
23. Transfer in the vapor phase of a liquid nitrogen tank the next day.

**Pause point:** PBMCs can be cryopreserved in liquid nitrogen for several years.

#### Thawing of PBMCs

**Timing: 30 min**

***Note:*** Ensure optimal recovery and viability of cells by strictly using pre-warmed (37°C) medium during thawing procedure.

24. Warm up PBMC culture medium to 37°C in a water bath before beginning with the thawing procedure.
25. Thaw PBMC cryovial at 37°C in a water bath by gently shaking the vial.

***Note:*** Do not submerge the entire cryovial completely under water.

**CRITICAL:** Avoid leaving the cryovial unattended while thawing, as the process typically completes within a minute or two. Remove when a small amount of approximately 20% ice crystal remains. Before opening, dry the outside of the cryovial and wipe with 70% alcohol to prevent contamination.

26. Add 500 μL PBMC culture medium to the cryovial.
27. Transfer PBMCs from the cryovial to a 50 mL centrifuge tube containing 1 mL of medium.
28. Drop-wise, add pre-warmed (37°C) PBMC culture medium into the 50 mL centrifuge tube containing PBMCs at a rate of 1 drop per second while swirling the tube until it is filled up to 32 mL.

***Note:*** Perform a serial dilution by first adding 2 mL, then 4 mL, 8 mL and finally 16 mL.

**CRITICAL:** Achieve optimal cell viability by gently rinsing cells with medium in a slow drop-wise manner, preventing any potential osmotic shock.

29. Centrifuge sample at 300 × *g* for 5 min at 20°C.
30. Aspirate the supernatant and resuspend the cells in 1 mL of pre-warmed (37°C) medium.
31. Perform a cell count by removing 10 μL for a Trypan Blue exclusion using a hemocytometer or automated cell counter.

***Note:*** Adhering to this protocol will result in a post-thaw cell viability exceeding 90%.

32. Culture in a T-25 (10 mL total volume) or T-75 flask (20 mL total volume) depending on the cell number and desired cell concentration (troubleshooting 4).

***Note:*** Within our laboratory we seed the cells at a density of 1–2 × 10^6^ cells/mL.

33. Incubate at 37°C with 5% CO_2_.

***Note:*** Prior to performing cell-based assays, it is advised that you rest the cells for 18–20 h.

### Preparing snap-frozen PBMC cell pellets for RNA and DNA extraction

**Timing: 20 min**

This section describes how to freeze down PBMC pellets, e.g., for isolation of RNA and/or DNA.

**CRITICAL:** Use RNase- and DNase- free microcentrifuge tubes.

34. After the cell count, pool the desired amount of cells (e.g., 2 × 10^6^ cells per aliquot) in a 15 mL centrifuge tube.
35. Fill it up with ice-cold DPBS.
36. Centrifuge at 720 × *g* for 5 min at 4°C.
37. Aspirate and discard the supernatant.
38. Fill up the tube with ice-cold DPBS.
39. Distribute 2 × 10^6^ cells in individual 1.5 mL microcentrifuge tubes.
40. Centrifuge at 720 × *g* for 5 min at 4°C.
41. Carefully remove the supernatant completely.

**CRITICAL:** Residual DPBS can affect RNA/DNA extraction and therefore diminish RNA/DNA quality.

42. Instantly snap-freeze the tube with the cell pellets by dipping in liquid nitrogen.
43. Store cell pellets at −80°C until further usage.

**Pause point:** PBMC pellets can be cryopreserved at −80°C for several years.

### DNA extraction from PBMC cell pellets for exome sequencing analysis

**Timing: ∼30 min**

This section describes how to isolate DNA from snap frozen PBMC pellets, containing ∼2 × 10^6^ cells by using the PureLink Genomic DNA Mini Kit (Thermo Fisher Scientific) according to manufacturer’s protocol https://tools.thermofisher.com/content/sfs/manuals/purelink_genomic_man.pdf with some adjustments.

44. Before you start with the DNA extraction protocol, reconstitute all the components supplied with the Kit according to the manufacturer’s recommendations.

***Note:*** Make sure there is no precipitate visible in the PureLink Genomic Digestion Buffer of PureLink Genomic Lysis/Binding Buffer (troubleshooting 5).

45. Set a water bath or a heat block to 55°C.
46. Add 20 μL Proteinase K to a sterile DNase free microcentrifuge tube.
47. Resuspend your pellet in 200 μL DPBS.
48. Transfer 200 μL cells in DPBS to the tube containing Proteinase K.
49. Add 20 μL RNase A to the sample.
50. Mix well by brief vortexing and incubate for 2 min at 20°C ± 2°C.

**CRITICAL:** To avoid shearing of DNA at each vortexing step, vortex your samples not more than 5–10 s.

51. Add 200 μL PureLink Genomic Lysis/Binding Buffer and mix well by vortexing to obtain a homogenous solution.
52. Incubate at 55°C for 10 min to promote protein digestion.
53. Add 200 μL 96%–100% EtOH to the lysate.
54. Mix well by vortexing to yield a homogenous solution.
55. Remove a PureLink Spin Column in a Collection Tube (supplied with the Kit).
56. Add the lysate (∼640 μL) prepared with PureLink Genomic Lysis/Binding Buffer and EtOH to the Spin column.
57. Centrifuge the column at 10.000 × *g* for 1 min at 20°C.

***Note:*** Perform all centrifugation steps at 20°C.

58. Discard the collection tube and place the spin column into a clean PureLink collection tube.
59. Add 500 μL Wash Buffer 1 prepared with EtOH to the column.
60. Centrifuge column at 10.000 × *g* for 1 min at 20°C.
61. Discard the collection tube and place the spin column into a clean PureLink collection tube.
62. Add 500 μL Wash Buffer 2 prepared with EtOH to the column.
63. Centrifuge the column at 21.130 × *g* (full speed) for 3 min at 20°C. Discard the collection tube.
64. Place the spin column in a sterile 1.5 mL microcentrifuge tube.
65. Add 50 μL of ultrapure water to the column.
66. Incubate at 20°C ± 2°C for 1 min.
67. Centrifuge the column at 21.130 × *g* (full speed) for 1 min at 20°C.

***Note:*** The tube contains purified genomic DNA.

68. Repeat steps 65–67 to recover more DNA.

**CRITICAL:** Second round of elution maximizes DNA recovery for ∼10%–15% gDNA. Use different tubes for the two-elution step to avoid contact of the spin column with the eluate.

69. Centrifuge the column at 21.130 × *g* (full speed) for 1.5 min at 20°C.

***Note:*** The tube contains purified genomic DNA.

70. Remove and discard the column.
71. Keep extracted DNA on ice.
72. Quantify genomic DNA using a Qubit fluorometric assay.
73. Store the purified DNA at 4°C for short-term or at −20°C long-term storage.

**Pause point:** Extracted DNA can be cryopreserved at −20°C for at least 24 months.

### Human colorectal tumor tissue

#### Production of RSPO-1 and Noggin conditioned media for organoid culturing

**Timing: 2–3 weeks**

***Note:*** R-spondin and Noggin conditioned medium are produced in-house using HEK293T cells that generate Rspo-I-Fc or Noggin-Fc. The cells are cultured for a period of 2–3 weeks to yield the proteins, a process extensively detailed in several publications.^2^^,^^3^^,^^4^

#### Tissue sampling, processing, and tumor organoid generation

**Timing: 3 h**

This section describes how to initiate organoid culture from patient-derived surgical specimens after the specimens were collected from the pathologist and transported back to the lab.

**CRITICAL:** To guarantee optimal maintenance of progenitor cell capacity *in vitro*, preservation of viability and successful organoid formation, the dissociation of tissue and isolation of cells should strictly be performed on fresh material only. Therefore, immediately proceed with sample processing after tissue collection and transportation.

74. Wash the tissue fragments 5× in ice-cold washing buffer by following the next steps:
  a. Pour the tissue with Tissue collection medium into a petri dish and keep on ice.
  b. Use sterile forceps to transfer the tissue into a 50 mL centrifuge tube containing 20 mL ice-cold washing buffer.
    ***Note:*** Use separate forceps and scalpels for healthy and tumor tissue.
  c. Shake vigorously for ∼10 s.
  d. Repeat steps a-c for 5× in total by using a fresh petri dish for every washing step.
  e. After the last washing step, continue with step 75.
    ***Note:*** Work fast and on ice to sustain cell viability.
75. Transfer tissue fragments to a petri dish.
76. Remove surrounding fat and connective tissue using a scalpel and forceps.
77. Weigh the specimens for documentation.
78. Mince the tissue with sterile scalpel and forceps < 5 mm^3^.
79. Transfer tumor pieces to a 50 mL centrifuge tube filled with 9 mL of Organoid digestion mix.
80. Rinse Petri dish with 1 mL of Organoid digestion mix and add to the 50 mL centrifuge tube.
81. Incubate for 1 h in a pre-warmed (37°C) Thermostable shaker at 180 rpm.
82. Stop digestion by adding 8 mL of StemPro medium + 2 mL FBS.

***Note:*** Fetal Bovine Serum (FBS) contains protease inhibitors which inhibit the enzymatic activity of the protein and therefore stops the digestion process.

**Figure 3:**
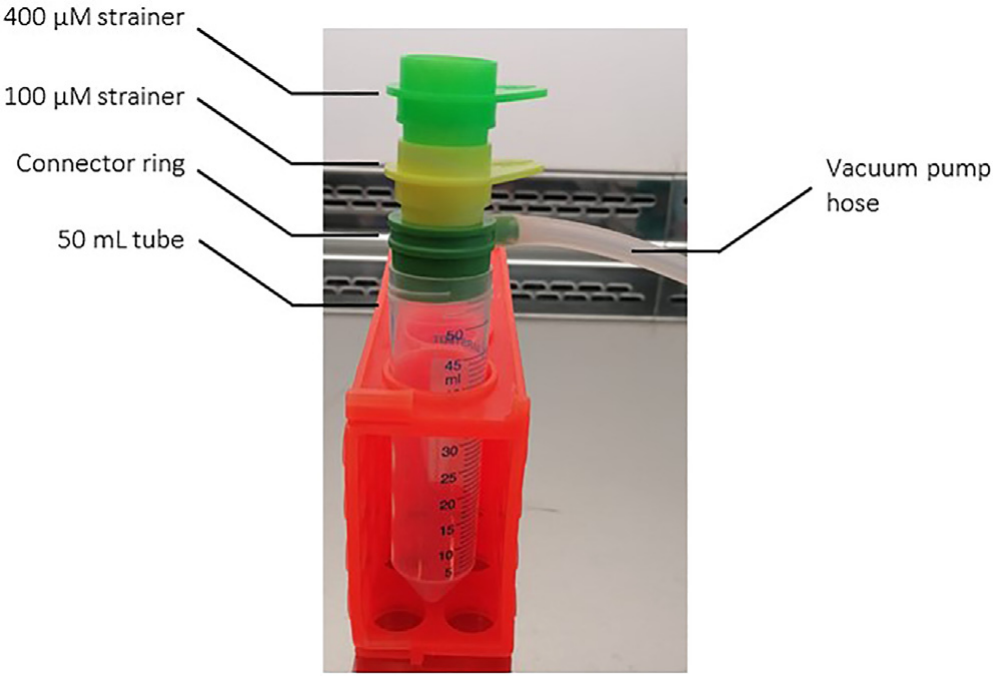
Setup for filtering cells through strainer for obtaining a single cell suspension after dissociation A 400 μM strainer (neon green) is placed on top of a 100 μM strainer (yellow) in the middle and a connector ring (dark green) on the bottom, attached to a 50 mL centrifuge tube connected to a vacuum pump.
84. Centrifuge at 200 × *g* for 4 min at 20°C.
85. Wash once with 20 mL of DPBS.
86. Centrifuge at 200 × *g* for 4 min at 20°C and carefully aspirate the supernatant.
87. Resuspend pellet with 3 mL 1× RBC-Lysis buffer.
88. Incubate for 10 min in the dark at 20°C ± 2°C .
89. Centrifuge 350 × *g* for 5 min at 20°C and carefully aspirate supernatant.
90. Wash 3× with 20 mL of DPBS.
91. Centrifuge at 200 × *g* for 3 min at 20°C and carefully aspirate supernatant.
92. Count cells manually in a Neubauer chamber (Hemocytometer).
93. Centrifuge at 200 × *g* for 5 min at 20°C and carefully aspirate supernatant.
94. Resuspend cells in appropriate amount of Advanced DMEM/F-12 to have 1.5 × 10^5^ cells in each 30 μL drop.
95. Add required volume of Geltrex to the cells (troubleshooting 6).

***Note:*** E.g., for each 30 μL droplet: 70% Geltrex + 30% Advanced DMEM/F-12, add 21 μL of Geltrex to 9 μL of cell suspension.

***Note:*** Do not press the pipette beyond the first stop before dispensing, to avoid bubble formation.

83. Collect dissociated cells in a 50 mL centrifuge tube by filtering through a layer of 400 μM strainer on top and 100 μM strainer below attached to a vacuum pump with a connector ring (Figure 3).

96. Seed 4 drops of 30 μL per well in a 6-well plate.
97. Let drops solidify for 30 min at 20°C ± 2°C or place in the incubator (37°C) for 15 min.
98. Add 2 mL of pre-warmed (37°C) Tumor organoid culture medium per well.

***Note:*** Ensure that the culture medium is not cold when you add it to the wells. Pre-warmed (37°C) medium or medium at 20°C ± 2°C better conserves Geltrex integrity.

***Note:*** Carefully add the medium by slowly pipetting on the side of the well and not directly on the drops to avoid disruption (troubleshooting 7).

99. Culture at 37°C and 5% CO_2_
100. Replace medium every 2–3 days.
101. Passage organoids after 5–10 days, depending on growth rate (see section “passaging, freezing and thawing of tumor organoids”).

***Note:*** Prior to reaching the second passage organoid cultures may contain residual debris from the isolation procedure.

### Passaging, freezing, and thawing of tumor organoids

**Timing: 30–60 min**

This section describes how to maintain organoid culture for expansion by weekly passaging and how to freeze and thaw samples for long-term storage in the vapor phase of a liquid nitrogen tank.

**CRITICAL:** Coat pipette tips and tubes with sterile 1% BSA in DPBS when splitting to avoid cells from sticking to plastic surfaces and minimize material loss.

#### Passaging of tumor organoids

**Timing: ∼30 min**

***Note:*** the splitting ratio depends on the individual proliferation rate of patient-derived organoids and will vary but as a rule of thumb split tumor organoids approximately every 3–4 days at a 1:6 to 1:8 ratio. Regular microscopic examination of the cultures is helpful to monitor organoid growth, morphological changes and accumulation of debris in the lumen and to ultimately ensure that passaging is carried out at optimal times.

102. Aspirate the medium and add 1 mL of pre-warmed (37°C) DPBS per well.
103. Transfer organoids to a 15 mL tube using a cell lifter.
104. Centrifuge at 300 × *g* for 5 min at 20°C.
105. Aspirate supernatant and incubate with 1 mL 1× Trypsin per 4 drops for 5–10 min in a water bath set at 37°C or incubator (37°C).
106. Add the same amount of medium to stop the reaction.
107. Use a 1 mL tip and pipet 10× up and down.
108. Centrifuge at 300 × *g* for 5 min at 20°C.
109. Aspirate supernatant completely and resuspend with desired amount of medium (30% of the total volume).

**CRITICAL:** When removing the medium in step 109 try to leave as little as possible residual liquid as it dilutes the Geltrex concentration and could increase the fragility of drops.

110. Put the vial on ice and add Geltrex (70% of the total volume) and mix gently.
111. Seed 4 drops of 30 μL per well in a 6-well plate.
112. Let drops solidify for 30 min at 20°C ± 2°C or place in the incubator (37°C) for 15 min.
113. Add 2 mL of tumor organoid culture medium per well.
114. Culture at 37°C and 5% CO_2_.

#### Freezing of tumor organoids

**Timing: ∼45 min Geltrex removal and freezing**

**CRITICAL:** It is beneficial to remove Geltrex before freezing because this will make seeding after thawing more scalable as you will have a more defined cell pellet and can control resuspension volume more precisely.

115. Remove the medium carefully without disturbing the Geltrex drops.
116. Wash wells with 1 mL of cold (4°C) DPBS.
117. Aspirate and add 1 mL of cold (4°C) Cell Recovery Solution.
118. Collect drops in a 15 mL centrifuge tube with a cell lifter.
119. Disrupt the drops by pipetting up and down several times until smaller fragments are visible.
120. Incubate for 30 min on ice or a shaker at 4°C.
121. Centrifuge at 300 × *g* for 5 min at 4°C.
122. Resuspend in 500 μL Recovery Cell Culture Freezing Medium per well of a 6-well plate.
123. Transfer 500 μL of the cell suspension to a cryovial and label accordingly.
124. Freeze at −80°C for 16–24 h in a Mr. Frosty freezing container.
125. Transfer in the vapor phase of a liquid nitrogen tank the next day.

**Pause point:** Tumor organoids can be cryopreserved in liquid nitrogen for several years.

#### Thawing of tumor organoids

**Timing: 30 min**

126. Place cryovial in a water bath (37°C) for appr. 2 min.
127. Add 500 μL of pre-warmed (37°C) Advanced DMEM/F-12 to the cryovial.
128. Without resuspending, transfer the cell suspension to a 15 mL centrifuge tube.
129. Fill up to 10 mL with Advanced DMEM/F-12 and centrifuge at 300 × *g* for 5 min at 20°C.
130. Aspirate supernatant and resuspend with desired amount of medium.
131. Put the vial on ice and add the required volume of Geltrex.
132. Seed 4 drops of 30 μL per well in a 6-well plate.
133. Let drops solidify for 30 min at 20°C ± 2°C or place in the incubator (37°C) for 15 min.
134. Add 2 mL of Tumor organoid culture medium per well.
135. Culture at 37°C and 5% CO_2_.

***Note:*** The number of organoids per drop is very important as too few or too many will affect the growth of the organoids. Before you plate all of your material, try with a small 10 μL drop first and check the density under the microscope. Start resuspending with a low volume and if the confluency is too high you can always adjust at this point and add more medium and Geltrex.

### Dissociation of tumor organoids into single cells

**Timing: 30–45 min**

This section describes how to generate single cells from tumor organoids with a high cell viability in order to perform downstream analysis e.g., single cell RNA sequencing.

**CRITICAL:** Organoids used for this protocol should not be too dense or contain dead cells. The morphology of the organoids needs to be considered as well, since an organoid with a cystic morphology contains cells only in the outer layer compared to an organoid with a budding morphology. Dissociation of 4 organoid culture drops leads to a cell yield which varies between 0.9–2.9 × 10^6^ single cells with a viability between 90%–97%.

136. Before starting the protocol, prepare the following reagents and switch on the following instruments:
  a. Warm up DPBS to 37°C.
  b. Keep Advanced DMEM/F-12 at 20°C ± 2°C.
  c. Freshly prepare 1× DPBS with 0.04% BSA and keep on ice.
  d. Prepare 1× DPBS with 1% BSA for coating the tips.
  e. Two centrifuges, one at 20°C, one at 4°C.

***Note:*** By coating tips with 1× DPBS with 1% BSA cell loss can be avoided.

137. Remove the medium in the wells.
138. Add 2 mL of pre-warmed (37°C) DPBS into the wells.
139. Scrape drops with a cell scraper.
140. Collect organoids in a 15 mL centrifuge tube.
141. Fill up to 10 mL with pre-warmed (37°C) DPBS.
142. Centrifuge at 405 × *g* for 5 min at 20°C.
143. Carefully discard supernatant.
144. Resuspend in pre-warmed (37°C) 1× Trypsin: use 500 μL for each drop used in the beginning.
145. Pipette 5× up and down with a P1000.
146. Incubate 5 min in a water bath set to 37°C.
147. Stop Trypsin-reaction by adding the same amount of Advanced DMEM/F-12 medium (22°C ± 2°C).
148. Mechanically dissociate the organoids by putting a 200 μL tip on top of a 1000 μL tip and pipette 10× slowly up and down.
149. Put cell suspension through a pre-wetted 40 μm cell strainer.
150. Collect the suspension in a 15 mL centrifuge tube.
151. Centrifuge at 405 × *g* for 5 min at 4°C.
152. Remove the supernatant carefully without disturbing the cell pellet.

***Note:*** Single cells should have formed a pellet.

153. Resuspend single cells with 1 mL of 1× DPBS with 0.04% BSA.
154. Vortex shortly and count manually using a hemocytometer with Trypan blue to assess viability.

**CRITICAL:** Keep dissociated single cells on ice during the counting process (troubleshooting 8).

### Isolation, culturing, and cryopreservation of tumor infiltrating lymphocytes (TILs) from human colorectal tissue

**Timing: 30 min to 1 h isolation, ∼2 weeks culture time, ∼30 min cryopreservation**

This section describes how to isolate tumor-infiltrating lymphocytes from human tissue fragments after surgical tumor resection. Keep tissue on ice during the transport from the pathologist to the lab.

#### Tumor infiltrating lymphocytes (TILs) isolation

**Timing: 30 min to 1 h**

155. Wash the tumor tissue 5× according to the washing steps in step 74 (see section ‘tissue sampling, processing and tumor organoid generation’).
156. After last washing step, remove fat and connective tissue by using a sterile scalpel and forceps.
157. Mince tumor tissue into small fragments (∼1 mm^3^ pieces).
158. Add 2 mL of pre-warmed (37°C) TILs isolation medium to the wells of a 24-well plate.
159. Put one tumor fragment (∼1 mm^3^) into each well of a 24-well plate using sterile forceps and scalpel (troubleshooting 9).

***Note:*** Tumor pieces settle onto the bottom of the well.

160. Add 1000 U/mL IL-2 into each well (use a new tip for each well).
161. Mix the medium containing IL-2 of each well by pipetting carefully 2× up and down.
162. Incubate at 37°C and 5% CO_2_.

***Note:*** Do not mince the tumor piece further when pipetting up and down.

#### Culturing of TILs

**Timing: ∼2 weeks culture time**

163. Change medium every second day by tilting the plate.
164. Carefully remove 1 mL of medium without touching the bottom of the plate.

***Note:*** The tissue with the extravasated cells is settled down to the bottom of the wells.

**Figure 4:**
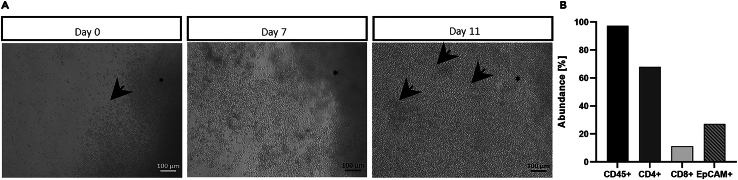
Isolation of tumor-infiltrating lymphocytes (TILs) by culturing tumor fragments (A) After 4 h (Day 0) of culturing tumor fragments (asterisks), TILs extravasate into the medium (arrow). After 11 days of culture, extravasated T cells form small clusters (arrows), which is a sign of T cell proliferation and activation. Images are acquired on an inverted light microscope (Zeiss). The black scale bars represent 100 μm. (B) Cell type distribution after two weeks of TILs isolation. Staining of isolated TILs with CD45^+^, CD8^+^, CD4^+^ and EpCAM^+^ antibodies. Samples are measured via Flow cytometry on a FACS Fortessa (BD) and analyzed by using FlowJo Software. Bar diagrams are generated using Graph Pad Prism software.

165. Add 1 mL of fresh pre-warmed (37°C) TILs isolation medium.
166. Add 1000 U/mL IL-2 into each well (use a new tip for each well).
167. Pipette up and down (use a new tip for every well).
168. Keep tumor fragments in culture for ∼ 14 days (Figure 4, troubleshooting 10).

#### Cryopreservation of TILs after ∼2 weeks of isolation

**Timing: ∼30 min**

**CRITICAL:** Before freezing down, make sure that the last medium change and the addition of IL-2 was ∼ 2 days ago, so that the cells are not in a stimulated state, since that would compromise the cell viability following freezing and thawing events.

169. Remove tissue fragments from the culture wells by using forceps.
170. Collect the cells by straining them through a 100 μm strainer to remove remaining tissue fragments.
171. Check the wells under a microscope and collect remaining cells by washing the wells with additional culture medium.

***Note:*** Pre-wet strainer with 1 mL of TILs isolation medium to avoid cell loss.

172. Centrifuge at 405 × *g* for 5 min at 20°C.
173. Remove supernatant and resuspend the cell pellet in 1 mL of pre-warmed (37°C) TILs isolation medium.

***Note:*** Keep cells on ice during counting.

174. Count the cells manually with a hemocytometer and assess viability by Trypan blue exclusion method.
175. Freeze 3–5 × 10^6^ cells in 1 mL of TILs cryopreservation medium.
176. Put cryotube(s) into Mr. Frosty freezing container at −80°C for 16–24 h and transfer into the N2 tank the next day for long-term storage.

**Pause point:** TILs can be cryopreserved in liquid nitrogen for several years.

***Note:*** This protocol describes TILs isolation from primary tumor tissue by culturing tumor fragments. Addition of IL-2 leads to extravasation of immune cells from the tissue. Amount of fragments cultured depends on the size of the tumor piece obtained from the pathologist. The difference in the range of TILs obtained after ∼2 weeks of culturing tumor fragments elucidates the need of an additional expansion protocol (see section ‘expansion of tumor infiltrating lymphocytes (TILs))’ in order to have enough starting material for downstream experiments.

***Note:*** Isolated TILs population contains EpCAM^+^ cells as well (Figure 4B).

***Optional:*** Subsequently a Fluorescence-Activated Cell Sorting (FACS) step of the cells of interest can be performed. E.g., CD45^+^ cells can be labeled with an anti-human CD45 antibody and sorted by using a FACS Aria Sorter (BD) and then used for further expansion. Especially when FACS is performed for more than 1 h, the cell viability can decrease. Use a FACS sorting medium with FBS, e.g., RPMI 1640 with the addition of 2 mM L-Glutamine, 1% Penicillin-Streptomycin and 1% FBS. Additionally a Live/Dead marker, e.g., 7-AAD can be included, to remove dead cells with the sorting process.

### Expansion of tumor-infiltrating lymphocytes (TILs)

**Timing: 1.5 h preparation, ∼2 weeks expansion time**

This section describes the expansion of TILs by using gamma irradiated (ɣ-irradiated) feeder cells. After ∼ 2 weeks of expansion, a high number of viable T cells are obtained, which can be used for downstream experiments.

#### Generation of feeder cells

**Timing: ∼45 min preparation, ∼15 min irradiation**

This section describes the mitotically inactivation of PBMCs from healthy donors (Blood Bank, Tirol Kliniken, Innsbruck, Austria) by ɣ-irradiation in order to use them as feeder cells for the expansion of the TILs.

177. One day before expansion, thaw PBMCs from healthy donors according to the thawing steps 24–33 (section ‘thawing of PBMCs’).
178. Culture them in PBMC culture medium in a T-75 flask.
179. Incubate for 18–20 h at 37°C with 5% CO_2_.
180. Next day, collect and transfer the PBMCs in their culture medium on ice to the clinics (Radiotherapy Medical University Innsbruck, Austria) for irradiation.
181. Irradiate PBMCs with 30 Gy.
182. After irradiation, transfer them on ice back to the lab and continue with the expansion protocol.

***Alternatives:*** If there is no access to a Radiotherapy unit and therefore ɣ-irradiation is not available, other common irradiation treatments can be used as well.^5^

#### TILs expansion protocol

**Timing: ∼30 min preparation, ∼2 weeks expansion time**

183. Put ∼ 0.4 × 10^6^ of T cells into a T-75 flask (upright) with 10 mL of pre-warmed (37°C) TILs expansion medium.
184. Add 30 ng/mL of anti-CD3 (OKT3), 3000 U/mL IL-2 and 100- fold more irradiated feeder cells.

***Note:*** By starting the expansion with 0.4 × 10^6^ T cells, 40 × 10^6^ feeder cells are added to the T cell suspension culture.

185. Pipette with a wide-bore serological pipette gently up and down.
186. Incubate the flask(s) at 37°C with 5% CO_2_.
187. Monitor the cells daily by observing the color of the expansion medium.

***Note:*** When the pH of the medium drops below pH 6.8, the pH indicator phenol red turns yellow, which means that the culture needs to be splitted because the nutrients within the culture medium are used up. Additionally when cell density increases, T cell clusters can be observed in the TILs expansion medium.

188. Split the expanded T cells by collecting them in a 50 mL centrifuge tube.
189. Centrifuge at 405 × *g* for 5 min at 20°C.
190. Remove the supernatant without disturbing the cell pellet.
191. Split 1:2 into new T-75 flasks in 10 mL of pre-warmed (37°C) TILs expansion medium and add 3000 U/mL IL-2.
192. Pipette gently up and down with a wide-bore serological pipette.
193. Incubate the flask(s) at 37°C with 5% CO_2_.
194. Monitor cells for ∼ 2 weeks and split the cells if needed according to the steps 188–193.
195. After ∼ 2 weeks, collect T cells and centrifuge them at 405 × *g* for 5 min at 20°C.
196. Count them manually by using a hemocytometer and assess viability using Trypan blue solution.
197. Freeze down 3–5 × 10^6^ cells/mL cryopreservation medium.
198. Put cryotube(s) into Mr. Frosty freezing container at −80°C for 16–24 h and transfer into the N2 tank the next day for long-term storage.

**Pause point:** Expanded TILs can be cryopreserved in liquid nitrogen for several years.

***Note:*** This protocol is also successfully used for T cell expansion using PBMCs.

### Preparation of organoid samples for proteomics/phosphoproteomics and transcriptomics analyses

**Timing: ∼2 h**

This section describes how to prepare frozen pellets of organoids for proteomics/phosphoproteomics and transcriptomics analyses (troubleshooting 11).

199. Extract the culture dish from the incubator and place it on ice.
200. Remove the cultivation medium from the wells.
201. Add 2 mL ice-cold DPBS per well.
202. Remove the DPBS from the wells.
203. Add 2 mL ice-cold DPBS per well.
204. Remove the DPBS from the wells.
205. Add 1 mL ice-cold Cell Recovery Solution (Corning) into each well of a 6-well plate.
206. Detach the droplets from the well using a cell lifter.
207. Collect the droplets in Cell recovery solution with P1000 and transfer to a 50 mL centrifuge tube.

***Note:*** Do not disrupt the droplets in the well by pipetting.

**Figure 5:**
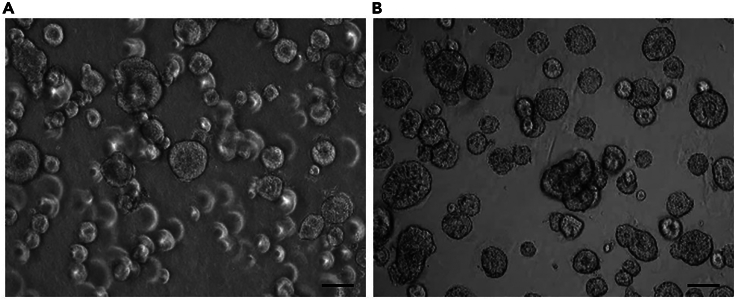
Tumor organoid appearance after Geltrex removal Images represent a tumor organoid culture derived from human colorectal cancer. (A) Image shows the organoids cultured in Geltrex droplets before Geltrex removal. (B) Image represents the same organoid culture after removal of Geltrex. Images are acquired on an inverted light microscope (Zeiss). The black scale bars represent 50 μm.

***Note:*** Do not remove the organoid pellet at the bottom of the tube.

208. Add 350 μL Cell Recovery Solution (Corning) into each well of a 6-well plate.
209. Collect the Cell recovery solution with a P1000 and transfer to the same 50 mL centrifuge tube.
210. Resuspend the droplets by pipetting 10× up and down in the 50 mL centrifuge tube using a P1000.
211. Incubate the 50 mL centrifuge tube for 30 min on ice.
212. Resuspend the droplets according to step 210.
213. Incubate the 50 mL centrifuge tube for 30 min on ice.
214. Centrifuge at 400 × *g* for 5 min at 4°C.
215. Remove the supernatant with a 5 mL serological pipet and any remaining Cell Recovery Solution with a P1000 tip (Figure 5).

216. Resuspend the pellet for 5× in 1 mL ice-cold DPBS using a P1000.
217. Fill the 50 mL centrifuge tube up with 25 mL ice-cold DPBS.
218. Centrifuge at 400 × *g* for 5 min at 4°C.
219. Remove the supernatant with a 5 mL serological pipette and any remaining DPBS with a P1000 tip.

***Note:*** Do not remove the organoid pellet at the bottom of the tube.

220. Resuspend the pellet for 5× in 1.5 mL ice-cold DPBS using a P1000.
221. Transfer the organoids solution into a 1.5 mL microcentrifuge tube.
222. Centrifuge at 400 × *g* for 5 min at 4°C.
223. Remove the supernatant with a P1000 tip and any remaining DPBS with a P100 and P10 tip.
224. Snap freeze the pellet by placing the 1.5 mL microcentrifuge tube in liquid nitrogen and store at −80°C until further use.

**Pause point:** Snap-frozen organoid pellets can be cryopreserved at -80°C for several years.

***Note:*** For proteomics/phosphoproteomics and transcriptomics analyses, directly use the frozen pellets. For protein extraction follow the appropriate protocol in accordance to the chosen downstream approach.

**CRITICAL:** Perform each step at 4°C. Ideally, perform every step in the cold room.

***Alternatives:*** In case a properly equipped cold room is not available, fill an icebox with ice and place a metal plate directly on the ice. Place the dish with organoids on the metal plate and perform steps 199–209. This will ensure to perform every step at 4°C while minimizing the risk of ice pieces falling into the wells.

#### Preparation of organoid samples for exome sequencing analysis

**Timing: ∼30 min**

This section describes how to prepare frozen pellets of organoids for exome sequencing analyses. Typically, for patient-derived CRC organoids, around 4 drops consisting of 30 μL of 70% Geltrex with confluent organoids are needed to generate 1 sample for exome sequencing analysis.

225. Extract the culture dish from the incubator.
226. Remove the cultivation medium from the wells.
227. Add 2 mL DPBS per well.
228. Remove the DPBS from the wells.
229. Add 2 mL DPBS per well.
230. Remove the DPBS from the wells.
231. Add 2 mL DPBS per well.
232. Detach the droplets from the well using a cell lifter.
233. Collect the droplets in DPBS with P1000 and transfer to a 15 mL centrifuge tube.
234. Add 350 μL DPBS well.
235. Collect the DPBS with a P1000 and transfer to the same 15 mL centrifuge tube.
236. Centrifuge at 400 × *g* for 5 min at 20°C.
237. Remove the supernatant with a P1000 tip.
238. Add 5 mL DPBS.
239. Centrifuge at 400 × *g* for 5 min at 20°C.
240. Remove the supernatant with a P1000 tip.
241. Add 1 mL DPBS.
242. Transfer the organoids solution into a 1.5 mL microcentrifuge tube.
243. Centrifuge at 400 × *g* for 5 min at 20°C.
244. Remove the supernatant with a P1000 tip.
245. Snap freeze the pellet by placing the 1.5 mL microcentrifuge tube in liquid nitrogen and store at −80°C until further use.

**Pause point:** Snap-frozen organoid pellets can be cryopreserved at −80°C for several years.

**CRITICAL:** Perform each step at 20°C ± 2°C, use DBPS pre-warmed at 20°C ± 2°C. To centrifuge the organoids efficiently, collect and centrifuge the droplets in 15 mL centrifuge tubes, avoid using 50 mL centrifuge tubes.

***Note:*** The preparation of pellets for exome sequencing analyses does not include a step of Geltrex removal. The DNA isolation performed with the protocol described in the following section is not influenced by the residual presence of Geltrex in the organoids frozen pellet.

### Isolation of DNA from organoids for exome sequencing analysis

**Timing: ∼30 min to 1 h**

This section describes the isolation of DNA using the PureLink Genomic DNA Mini Kit (Thermo Scientific) according to manufacturer’s protocol https://tools.thermofisher.com/content/sfs/manuals/purelink_genomic_man.pdf optimized for frozen organoid pellets by following the steps 44–73 (see section ‘DNA extraction from PBMC cell pellets for transcriptomic analysis’) with the following adjustments. Subsequently perform a cleanup step by using the Genomic DNA Clean & Concentrator Kit (Zymo Research) according to manufacturer’s protocol https://files.zymoresearch.com/protocols/_d4010_d4011_genomic_dna_clean_concentrator-10.pdf with the following adjustments.

**CRITICAL:** Perform each step at 20°C ± 2°C. Prepare Wash Buffer 1 and Wash Buffer 2 provided in the PureLink Genomic DNA Mini Kit (Thermo Scientific) using ≥ 99.8% ethanol following manufacturer's instructions.

#### Isolation of DNA using the PureLink genomic DNA Mini Kit (Thermo Scientific)

246. Adjust step 65 (see section ‘DNA extraction from PBMC cell pellets for exome sequencing analysis’) by adding 25 μL of ultrapure water to the column.
247. Incubate for 1 min at 20°C.
248. Centrifuge the column at 21.000 × *g* for 1 min at 20°C.

***Note:*** The microcentrifuge tube contains purified genomic DNA. Use the same tube for the second centrifugation.

249. Add 25 μL of ultrapure water to the same column.
250. Incubate for 1 min at 20°C.
251. Centrifuge the column at 21.000 × *g* for 1 min at 20°C.
252. Remove and discard the column.

***Note:*** The microcentrifuge tube contains purified genomic DNA.

253. Measure the concentration and purity of the DNA at OD 260/230 and 260/280 using a Spectrophotometer.

**Pause point:** Extracted DNA can be cryopreserved at −20°C for at least 24 months.

**CRITICAL:** Store the isolated DNA at 4°C for short-term storage up to 2 days, or at −20°C for long-term storage. Avoid repeated freezing and thawing.

***Note:*** Make sure to load the solutions (lysates, buffer or elution solution) onto the center of the column directly on the column membrane without touching or disrupting the membrane with the pipet tip.

***Note:*** DNA samples can additionally be purified and concentrated by using the following ‘Concentration and purification of DNA with the Genomic DNA Clean & Concentrator’ protocol.

#### Concentration and purification of DNA with the genomic DNA Clean & Concentrator (Zymo Research)

**CRITICAL:** Perform each step at 20°C ± 2°C. Prepare DNA Wash Buffer PureLink Genomic DNA the Genomic DNA Clean & Concentrator (Zymo Research) using ≥ 99.8% ethanol following manufacturer's instructions.

254. Add 200 μL of ChIP DNA Binding Buffer to the microcentrifuge tube containing 100 μL of DNA and resuspend 5× with a P1000 to obtain a homogenous solution.
255. Transfer mixture (300 μL) to a provided Zymo-Spin IC-XL Column2 placed in a collection tube (supplied with the kit).
256. Centrifuge the column at 10.000 × *g* for 30 s at 20°C.
257. Discard the flow through and place the column back in the same collection tube.
258. Add 200 μL DNA Wash Buffer to the column.
259. Centrifuge the column at 10.000 × *g* for 1 min at 20°C.
260. Discard the flow through and place the column back in the same collection tube.
261. Add 200 μL DNA Wash Buffer to the column.
262. Centrifuge the column at 10.000 × *g* for 1 min at 20°C.
263. Discard the collection tube and place the column in a sterile 1.5 mL microcentrifuge tube.
264. Add 15 μL of ultrapure water to the column.
265. Incubate for 1 min at 20°C ± 2°C.
266. Centrifuge the column at 15.000 × *g* for 30 s at 20°C.
267. Remove and discard the column.

***Note:*** The microcentrifuge tube contains purified genomic DNA.

268. Measure the concentration and purity of the DNA at OD 260/230 and 260/280 using a Spectrophotometer.

**Pause point:** Extracted DNA can be cryopreserved at −20°C for at least 24 months.

***Note:*** Make sure to add the DNA solution, the Wash Buffer and the ultrapure water onto the center of the column directly on the column membrane without touching or disrupting the membrane with the pipet tip.

### Isolation of RNA from organoids for transcriptomics analysis

**Timing: ∼30 min**

This section describes the isolation of RNA using the RNeasy Plus Mini Kit (QIAGEN), the QIAshredder (QIAGEN) and the RNase free DNase set (QIAGEN) according to manufacturer’s protocol https://www.qiagen.com/us/resources/resourcedetail?id=1d882bbe-c71d-4fec-bdd2-bc855d3a4b55&lang=en optimized for snap-frozen organoid cell pellets. Follow the instructions in the section “preparing snap-frozen PBMC cell pellets for RNA and DNA Extraction” to make snap-frozen organoid cell pellets, used as a starting material.

269. Before you start with the RNA extraction protocol, prepare the following reagents according to the manufacturer’s recommendations:
  a. Prepare Buffer RPE contained in the RNeasy Plus Mini Kit (QIAGEN) using ≥ 99.8% ethanol.
  b. Prepare a 70% ethanol stock solution using ≥ 99.8% ethanol and sterile H_2_O.
  c. Solubilize the DNase I of the RNase free DNase set (QIAGEN) buffer.

**CRITICAL:** Perform each step at 20°C ± 2°C. Add β-mercaptoethanol to the necessary amount of RLT buffer following manufacturer's instructions and use it immediately.

270. Transfer microcentrifuge tube containing frozen organoid cell pellet from −80°C into liquid nitrogen.
271. Proceed immediately while the pellet is still frozen.
272. Add 350 μL of Buffer RTL supplemented with β-mercaptoethanol to the pellet.
273. Resuspend with a P1000 until the pellet dissolves completely.
274. Homogenize the lysate by directly transferring it into a QIAshredder spin column placed in a 2 mL collection tube.
275. Centrifuge at 21.130 × *g* (full speed) for 2 min at 20°C.
276. Discard the column.

**CRITICAL:** The collection tube contains the homogenized lysate.

277. Add 350 μL of 70% ethanol to the homogenized lysate in the collection tube, resuspend 5× with a P1000 to obtain a homogenous solution.

***Note:*** Resuspend the ethanol-lysate mix well by pipetting, but don’t vortex.

278. Transfer the mixture (∼700 μL) into a RNeasy Mini spin column placed in a 2 mL collection tube (supplied with the kit).
279. Centrifuge at 10.000 × *g* for 30 s at 20°C.
280. Discard the flow through and place the column back in the same collection tube.
281. Add 350 μL of Buffer RW1 to the column.
282. Incubate the RW1 buffer for 5 min on the column at 20°C ± 2°C.
283. Invert tube and centrifuge at 10.000 × *g* for 30 s at 20°C.

**CRITICAL:** By inverting the tube, the membrane of the column is washed thoroughly.

284. Discard the flow through and place the column back in the same collection tube.
285. Add 10 μL DNase I stock solution to 70 μL Buffer RDD.
286. Add 80 μL of DNase I mix to the column.
287. Incubate for 15 min at 20°C ± 2°C.
288. Add 350 μL Buffer RW1 to the column.
289. Centrifuge at 10.000 × *g* for 30 s at 20°C.
290. Discard the flow through and place the column back in the same collection tube.
291. Add 500 μL of Buffer RPE to the column.
292. Centrifuge at 10.000 × *g* for 30 s at 20°C.
293. Discard the flow through and place the column back in the same collection tube.
294. Repeat steps 291 and 292.
295. Discard the flow through and place the column into a new collection tube (supplied with the kit).
296. Centrifuge at 10.000 × *g* for 2 min at 20°C.
297. Discard the collection tube and place the column in a sterile 1.5 mL microcentrifuge tube (supplied with the kit).
298. Centrifuge at 21.130 × *g* (full speed) for 1 min at 20°C.
299. Add 30 μL of RNase-free H_2_O to the column.
300. Incubate for 5 min at 20°C ± 2°C.
301. Centrifuge at 21.130 × *g* (full speed) for 1 min at 20°C.
302. Remove and discard the column.

***Note:*** The microcentrifuge tube contains purified RNA.

303. Keep purified RNA on ice.
304. Measure the concentration and purity of the RNA at OD 260/230 and 260/280 using a Spectrophotometer, RNA fragmentation using a Bioanalyzer and quantify genomic DNA using a Qubit fluorometric assay.
305. Store extracted RNA at −80°C until further use.

**Pause point:** Extracted RNA can be cryopreserved at −80°C for several years.

**CRITICAL:** Make sure to add the DNase I mix onto the center of the column directly on the column membrane without touching or disrupting it, to ensure complete DNase digestion.

**CRITICAL:** Make sure to add the RNase-free H_2_O onto the center of the column directly on the column membrane to ensure full membrane hydration and RNA elution.

### Human colorectal healthy tissue

#### Tissue sampling, processing, and healthy organoid generation

**Timing: 2 h**

***Note:*** For tissue sampling and processing follow steps 74–78 (see section ‘tissue sampling, processing and tumor organoid generation’).

306. Transfer the minced tissue into a 15 mL centrifuge tube filled with 10 mL of ice-cold 2 mM EDTA in DPBS and incubate on a rotator for 1 h at 4°C.
307. Allow the tissue fragments to settle down and remove the supernatant.

***Note:*** Work on ice to sustain cell viability.

**Figure 6:**
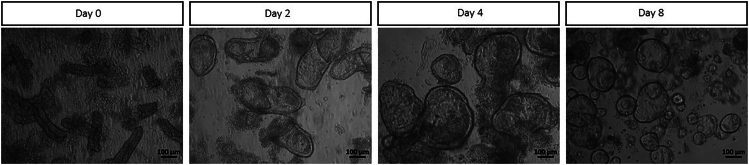
Representative images of different stages of healthy Organoid generation and culture over time Day 0 shows crypts seeded in a Geltrex drop directly after isolation followed by day 2, 4 and 8 in culture. Images are acquired on an inverted light microscope (Zeiss). The black scale bars represent 100 μm.

308. Vigorously pipet tissue pieces in 4 mL of DPBS 20× up and down with a P1000 and collect crypts in a new 15 mL centrifuge tube with 1 mL of FBS.
309. Repeat step 308 two more times.
310. Centrifuge the 15 mL centrifuge tube containing the harvested crypts in DPBS at 115 × *g* for 3 min at 4°C.
311. Aspirate the supernatant and count crypts by making a 10 μL drop in a petri dish and counting under a bright-field microscope using a 10× objective.
312. For each 30 μL drop seed 600 crypts (Figure 6, troubleshooting 12).

#### Passaging of healthy organoids

**Timing: ∼30 min**

***Note:*** The splitting ratio depends on the individual proliferation rate of patient-derived organoids and will vary but as a rule of thumb split healthy organoids approximately once a week at a 1:2 to 1:4 ratio (troubleshooting 13).

313. Aspirate the medium and add 1 mL of pre-warmed (37°C) DPBS per well.
314. Transfer organoids to a 15 mL centrifuge tube using the cell lifter.
315. Centrifuge at 300 × *g* for 3 min at 20°C.
316. Aspirate supernatant and incubate with 1 mL Accutase per 4 drops for 3–5 min in water bath set to 37°C or incubator (37°C).
317. Inactivate Accutase by adding the same amount of medium.
318. Put a 200 μL tip over a 1000 μL tip and pipette up and down for appr. 20 times.

**CRITICAL:** Monitor fragmentation of organoids under the microscope and if needed continue with mechanical splitting until there are no remaining intact organoids visible under the microscope. This step is crucial to ensure successful expansion. If organoids remain intact you will not gain any new material. Ideally, cell clumps need to be obtained before seeding but strictly avoid dissociating so much that you only have single cells left (troubleshooting 14).

319. Centrifuge at 300 × *g* for 3 min at 20°C.
320. Aspirate supernatant completely and resuspend with desired amount of medium.
321. Put the vial on ice and add Geltrex.
322. Seed 4 drops of 30 μL per well in a 6-well plate.

***Note:*** Alternatively, we recommend seeding 10 μL Geltrex domes (in total 120 μL per well of a 6-well plate) for improved growth rate.

323. Let drops solidify for 30 min at 20°C ± 2°C or place in the incubator (37°C) for 15 min.
324. Add 2 mL of Healthy organoid culture medium per well.
325. Culture at 37°C and 5% CO_2_.

***Note:*** For cryopreservation and thawing, follow steps 115–135 (see section ‘freezing of tumor organoids’ and section ‘thawing of tumor organoids’).

**Pause point:** Healthy organoids can be cryopreserved in liquid nitrogen for several years.

### Dissociation of healthy and tumor colorectal tissue into single cells

**Timing: 2–2.5 h**

This section describes in detail how to dissociate healthy and tumor colorectal tissue into viable single cells, which downstream can be used for molecular analysis, e.g., single cell RNA sequencing, flow cytometry measurements, etc.

***Note:*** Prepare media and reagents needed (see section ‘preparation of sample collection’).

***Note:*** Work under sterile conditions and keep tissue samples on ice at all times.

***Note:*** Do not add FBS to the medium, because downstream it interferes with RNA sequencing.^6^

326. Process and wash tissue according to the washing steps 74–78 (see section ‘tissue sampling, processing and tumor organoid generation’).
327. Prepare 10 mL of Tissue digestion mix (2×) and transfer it into a gentleMACS C tube.

***Note:*** For tissue ≤ 2 g use 10 mL of Tissue digestion medium. Adjust volume of the digestion mix according to the tumor weight.

**CRITICAL:** Working fast and on ice is essential for a viable single cell dissociation. Use different scalpels and forceps for healthy and tumor tissue otherwise tissues can be contaminated.

328. Transfer tissue pieces into gentleMACS C tubes containing the digestion medium.
329. Tightly close the C tubes and attach them upside down onto the sleeve of the gentleMACS dissociator.
330. Put the heaters on top of the gentleMACS C tubes.

***Note:*** The gentleMACS heaters are needed for the combined enzymatic and mechanical dissociation at 37°C.

**CRITICAL:** Make sure, no tissue piece is attached to the wall of the falcon. For an optimal dissociation, tissue pieces should be in the digestion medium.

331. Select the appropriate dissociation program for your species and organ of choice (tumor type:soft, program: 37C_h_TDK_1).

***Note:*** You can also customize dissociation programs according to the manufacturer’s protocol. If you work with tumors, consider that tumor tissue type can be distinguished from soft, to medium, to tough. Different programs with different durations and steps are used based on the tumor type.

332. Stop the program after 45 min at 37°C.

***Note:*** Check if tissue pieces are completely dissociated. If not, proceed with the program.

333. Detach C tube from the dissociator.
334. Centrifuge at 300 × *g* for 30 s at 20°C.
335. Remove the supernatant without disturbing the cell pellet.

**CRITICAL:** If cells can be observed in the supernatant after centrifugation, centrifuge again at 300 × *g* for max. 3 min at 20°C by switching on the deceleration (set to 7) of the centrifuge in order to gently stop the centrifuge, to collect all the cells without loss of material.

***Note:*** Use a P1000 to remove the supernatant instead of a vacuum pump, since the cell pellet might not be very firm.

336. Add 10 mL Tissue digestion medium and gently resuspend the cell pellet.
337. Filter the cell suspension through a pre-wetted 100 μm strainer placed on a 50 mL centrifuge tube.

***Note:*** After being pre-wetted, aqueous solutions can more easily pass through a hydrophilic membrane with reduced loss of material.

338. Wash the 100 μm strainer by passing again 10 mL Tissue digestion medium through and collecting the wash in the 50 mL centrifuge tube with the cell suspension.
339. Centrifuge cell suspension at 300 × *g* for 7 min at 20°C.
340. Aspirate supernatant completely.
341. Perform Red blood cell lysis by using Red Blood Cell Removal Solution.

**CRITICAL:** Prolonged exposure to Red Blood Cell Removal Solution could lead to an unintended tumor cell lysis.

342. Add 3 mL chilled 1× Red Blood Cell Removal Solution to the cell pellet.
343. Gently resuspend the cells.

***Note:*** Do not vortex.

**Figure 7:**
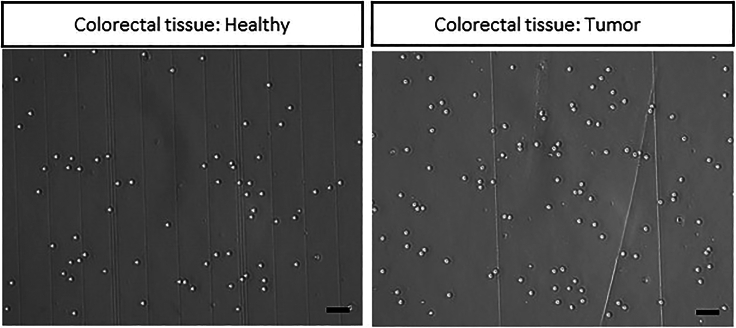
Healthy and tumor colorectal tissue dissociation into single cells Healthy and tumor CRC tissue after enzymatic and mechanical dissociation into single cells. Images are acquired on an inverted light microscope (Zeiss). The black scale bars represent 20 μm.

344. Incubate for 10 min at 20°C ± 2°C.
345. Add 10 mL chilled DPBS with 0.04% BSA.
346. Centrifuge at 300 × *g* for 10 min at 4°C.
347. Remove supernatant without disturbing the cell pellet.
348. Wash again, by adding 5 mL of DPBS with 0.04% BSA.
349. Centrifuge at 300 × *g* for 10 min at 4°C.
350. Remove supernatant and count cells manually with a hemocytometer and assess viability by using Trypan Blue solution (Figure 7).

**CRITICAL:** Keep single cell suspension on ice after red blood cell lysis and during cell counting to avoid decreased cell viability (troubleshooting 15).

## Expected outcomes

This protocol describes the processing of different specimens derived from human colorectal cancer tissue. We generated tumor and healthy organoid cultures and prepared tumor organoid samples for RNA-, DNA extraction, (Phospho-) proteomics and scRNA-seq analysis. The expected RNA yield after purification ranges from 320 to 1000 ng/μL for 4 drops of confluent tumor organoids. The estimated ratio for nucleic acid purity, measured at 260 nm and 280 nm wavelength (A_260_/A_280_), is 2.07–2.10. A A_260_/A_280_ ratio of ∼2.0 is accepted as pure RNA. The estimated RNA integrity has a RIN value of > 9. High-quality RNA contains a RIN value of ≥ 8 (max. RIN value is 10). The percentage of contamination with genomic DNA is ∼ 3%, which indicates almost no contamination. The expected DNA yield after purification ranges from 30–120 ng/μL for 4 drops of confluent tumor organoid. The A_260_/A_280_ ratio for nucleic acid purity is 1.8–2.0. The percentage of RNA contamination is < 4 ng/μL, which indicates almost no contamination. Dissociation of 4 confluent drops of tumor organoids into single cells yields in 1–3.5 × 10^6^ single cells with a viability between 90%–97%. In order to perform phosphoproteomics analysis around 48 confluent drops of patient-derived tumor organoids consisting of 30 μL of 70% Geltrex are needed to generate one sample for phosphoproteomics analysis. This corresponds to a ∼1 mg protein sample. For proteomics analysis, six drops of confluent tumor organoid drops are required.

TILs were isolated by using tumor fragments. From a tumor piece ≤ 2 g , ∼12–17 tumor fragments (1 mm^3^) can be cultured. After two weeks of TILs isolation, the cell yields from ∼2–30 × 10^6^ cells with a viability > 85%. The viability will increase after subsequent expansion to > 95%.

We isolated PBMCs from autologous blood samples. Expected PBMC yield from adult whole blood ranges from 1.2–3.5 × 10^6^ cells/mL. The expected DNA yield after purification ranges from 30–60 ng/μL for pellets consisting of ∼2 × 10^6^ cells. The estimated A_260_/A_280_ ratio is 1.78–1.90. A A_260_/A_280_ ratio of ∼1.8 is accepted as pure DNA. The percentage of RNA contamination is < 4 ng/μL, which indicates almost no contamination.

Dissociation of healthy and tumor colorectal tissues (≤ 2 g) yields in 6–9 × 10^6^ single cells with a viability > 95%. We did not observe any correlation between tissue weight and the number of single cells obtained after dissociation.

By following this protocol, generated organoids with their autologous PBMCs and TILs can further be used to perform co-culture experiments.^7^^,^^8^ Additionally, you can generate and sustain a culture of tumor and healthy colon organoids derived from surgical samples over an extended time period and expand them for biobanking. Accurate execution of the protocol is crucial for maintaining a high viability and purity of cells for various down-stream experimental applications.

## Limitations

The main limitation of this protocol lies in the accessibility and quality of the tissues. In our experience, a minimum weight of 0.3 g of tissue is sufficient to generate organoids. Preparing samples especially for phosphoproteomics analysis is limited by the excessive amount of input material needed for preparing samples, in order to meet the need for replicates required to perform statistical analysis. Using organoids as a model system has certain limitations which need to be considered as well. Organoids derived from adult stem cells only consist of epithelial cells and lack stromal and immune cells. Besides, unlike in the human intestine, the apical surface of the epithelial cells is facing the inside of the lumen and the basal side is facing the exterior side instead. If experiments require contact to the apical side, the polarity of the organoids has to be switched^9^ or microinjection has to be performed. Moreover, growing them in a 2D-monolayer or breaking them apart prior to incubation with cells or drugs provides alternative strategies to circumvent this spatial entity.

In contrast to more advanced methodologies, the density gradient centrifugation employed in this protocol is labor- and time-intensive and lacks automation feasibility. The delicate process of layering blood over the gradient demands patience and precision to prevent catastrophic mixing and disturbance. Inadequate layering risks partial or complete loss of target cells. Extracting the buffy coat, located at the medium-plasma interface, is another critical step prone to contamination. Contamination of harvested cells with other layers is hard to avoid but should be kept minimal since the gradient medium leads to osmotic stress which can impact cell viability. Overall, this method demands meticulous execution to mitigate potential pitfalls and yield reliable results.

## Troubleshooting

### Problem 1

Paragraph ‘Isolation of PBMCs from whole blood’: PBMC yield is rather low after isolation.

### Potential solution

The protocol works best when using blood within 2–3 h after blood collection. Moreover, all the required reagents must be at 20°C ± 2°C before using. A certain degree of donor-to-donor variability in cell yield is expected.

### Problem 2

Step 5: Insufficient formation of layers or separation medium and blood have mixed.

### Potential solution

Ideally fresh blood should be used for this protocol and coagulation of the whole blood should be prevented by utilizing EDTA tubes, ensuring proper agitation, and storing it at 20°C ± 2°C. To facilitate a gradual layering of whole blood onto the separation medium, decrease the ejection speed of the Pipetboy and tilt the tube. This technique aids in achieving a slow and controlled layer of the blood onto the separation medium. It is critical to centrifuge immediately, otherwise layers will gradually blend.

### Problem 3

Paragraph ‘Cryopreservation and subsequent thawing of PBMCs’: Low cell recovery of PBMCs following thawing.

### Potential solution

During the cryopreservation process, prioritize speed and efficiency, working promptly to preserve cell integrity. Strictly work on ice uninterruptedly and make sure your freezing medium has the correct composition. Also prevent cells from lingering in the freezing medium at 20°C ± 2°C post-re-suspension. Utilize Mr. Frosty freezing container adequately to freeze cells gradually at a rate of −1 °C/min and make sure it is filled with the required volume of Isopropyl for optimal cryopreservation results. Maximum cell recovery and viability can be achieved by strictly working with pre-warmed medium throughout the entire thawing process. A serial dilution and dropwise addition is recommended to avoid any potential osmotic shock. Human primary cells are very fragile to handle so guarantee to handle the cells gently when pipetting by using wide-bored pipettes if possible. Minimize excessive pipetting to prevent potential damage to cells.

### Problem 4

Step 32: PBMCs are clumping heavily.

### Potential solution

Filter PBMCs through a 70 μm cell strainer to remove debris and bigger cell clumps. Add 0.04% BSA (400 μg/mL) into your 1× DPBS to prevent cell clumps. Dying cells release DNA into the media, which can lead to clumping as well. DNase I at 200 U/mL can be added into the pre-warmed medium when thawing cells and incubated for 15 min at 20°C ± 2°C. This intervention might help to reduce the tendency for cells to stick together.

### Problem 5

Step 44: Precipitates are visible in the Digestion or/and Lysis/Binding Buffer of the PureLink Genomic DNA Mini Kit.

### Potential solution

Warm the buffers up at 37°C for 3–5 min. Check, if no precipitations are visible after 5 min and mix well before use.

### Problem 6

Step 95: Geltrex is too liquid or too solidified while seeding Geltrex drops.

### Potential solution

Ensure that the Geltrex is thoroughly thawed on ice before using. It is essential to guarantee a homogenous mix of Geltrex and medium and to hinder the polymerization process prior to seeding. Pipet tips can be stored in the freezer before usage and well plates can be pre-warmed in the incubator at 37°C.

### Problem 7

Step 98: Geltrex drop integrity is disrupted.

### Potential solution

Probably the Expansion medium was added before the Geltrex drop was completely polymerized. Next time make sure you wait long enough and add the medium carefully, ensuring that it is not added directly on a drop but on the side of the well.

### Problem 8

Step 154: Low viability after dissociation of tumor organoids into single cells.

### Potential solution

Splitting ratio is important and should be optimized before organoid dissociation into single cells. Select organoid drops from different wells, if you have expanded more wells in order to avoid using the drops which are too dense or contain dead cells.

### Problem 9

Step 159: Contamination of tumor fragments.

### Potential solution

In order to prevent contamination use sterile instruments (scalpels and forceps) and work under sterile conditions. Performing washing steps with a washing buffer containing antibiotics (Penicillin/Streptomycin and Primocin) will help to prevent contaminations as well. Make sure that the resected colorectal tissue piece is cut and washed out properly before transported to the pathologist, which reduces the probability of contamination too.

### Problem 10

Step 168: Cell yield of TILs is quite low after two weeks of expansion.

### Potential solution

This expansion protocol can be extended to three weeks, but the culture needs to be monitored closely. Change of the color of the medium and too many cell clumps are signs that the culture needs to be splitted. Avoid splitting in a higher ratio than 1:2, because T cells need a certain density in order to proliferate properly.

### Problem 11

Paragraph ‘Preparation of organoid samples for proteomics/phosphoproteomics and transcriptomics analysis’: The organoids pellets provide an insufficient amount of protein or RNA for downstream analysis.

### Potential solution

The amount of organoid material specified in this protocol are indicative and related to human colorectal cancer patient-derived organoids. It is advisable to run pre-experiments to assess the amount of organoid material required for each preparation in accordance with the requirement for your specific downstream omics analyses approaches. Organoids derived from different tissues as well as different patients might have a different yield.

### Problem 12

Step 312: After seeding hardly any or no healthy organoids form.

### Potential solution

Optimize the seeded cell/crypt density and ensure that ROCK inhibitor is added at a final concentration of 10 μM when passaging.

For healthy Organoids, do not allow EDTA incubation to exceed 1.5 h and wash thoroughly.

When you harvest the crypts, pipet them carefully so they don’t break but remain intact.

### Problem 13

Paragraph ‘Passaging of healthy organoids’: Organoids lose structural integrity over culture time and start to look like adherent cells grown in 2D.

### Potential solution

Organoids might have been sinking down and attached to the bottom of the well. This is usually due to insufficient matrix to support the cells in the organoid. Typically, Geltrex needs replacing as it has formed a concentration gradient over long periods of time. Alternatively, invert the Geltrex domes containing the organoids during incubation to prevent the organoids from settling at the bottom of the well.

### Problem 14

Step 318: Inconsistent size / diameter of organoids in same drop.

### Potential solution

Organoids need to be more vigorously and uniformly disrupted during passaging by pipetting or incubated longer with Accutase or in higher volume. Check under the microscope and if necessary, repeat the procedure.

### Problem 15

Step 350: Decreased viability after tissue dissociation into single cells.

### Potential solution

Working on ice and fast prevents a decrease of viability. Mincing the tissue in small pieces before dissociation is a critical step, where rigorous mincing leads to reduced viability. The tissues are dissociated during mechanical dissociation as well, so an excessive mincing is not necessary. Working fast and monitoring the tissue or cells at every step provides a lot of information. After dissociation, single cells need to be handled gently, an excessive resuspension after every centrifugation step is not recommended.

## Resource availability

### Lead contact

Further information and requests for resources and reagents should be directed to and will be fulfilled by the lead contact, Zlatko Trajanoski (zlatko.trajanoski@i-med.ac.at).

### Technical contact

Further information and requests for resources and reagents should be directed to and will be fulfilled by the technical contact, Zlatko Trajanoski (zlatko.trajanoski@i-med.ac.at).

### Materials availability

This study did not generate new unique reagents.

### Data and code availability

This study did not generate new unique datasets or codes.
